# Pairwise Comparison of Effects of Linear vs. Change of Direction Short Bout Sprint Intervals on Physical Performance of Youth Male Soccer Players

**DOI:** 10.3390/sports14020044

**Published:** 2026-01-26

**Authors:** Peter Sagat

**Affiliations:** Sport Sciences and Diagnostics Research Laboratory, GSD/Health and Physical Education Department, Prince Sultan University, Riyadh 11586, Saudi Arabia; sagat@psu.edu.sa

**Keywords:** football, repeated sprint ability, linear sprinting, change of direction, effects

## Abstract

Our study aimed to examine and compare the effects of 12-week repeated sprint intervals with change of direction and linear sprint intervals on physical performance in young soccer players. In this randomized, parallel three-group study, we included 60 male soccer players assigned to (i) a sprint interval with change of direction group (RS–CoD; n = 20); (ii) a linear sprint interval group (RS–LiN; n = 20); and (iii) a soccer group (SOC; n = 20). Physical performance included explosive power (countermovement jump [CMJ] and squat jump [SJ]), agility (T505, 93,639, 20Y), speed (sprints over 5 m, 10 m and 20 m), anaerobic capacity (the Running-Based Anaerobic Sprint Test [RAST]) and maximal oxygen uptake (VO2max). Over the 12 weeks, the RS–CoD group displayed significantly beneficial effects in the 93639 test (effect size [ES] = 0.42), compared to the RS–LiN (ES = 0.18) and SOC (ES = 0.12) groups. The RS–CoD group also had larger improvements in their SJ (ES = 0.87; RS–LiN 0.37; SOC 0.18), CMJ (ES = 0.56; RS–LiN 0.39; SOC 0.43), 20Y test (ES = 1.05; RS–LiN 0.67; SOC 0.56) and sprints at 5 m (ES = 1.18; RS–LiN 0.50; SOC 0.21) and 20 m (ES = 1.43; RS–LiN 0.71; SOC 0.25). The RS–CoD group displayed more beneficial improvements, making the CoD interval sprints effective training stimuli.

## 1. Introduction

High-intensity actions like sprints and vast changes of direction (CoDs) performed at maximal speeds exceeding 19.5 km*h^−1^ have become crucial for achieving an advantage over the opponent [1]. Irrespective of playing position, evidence suggests that between 4% and 18% of the total distance covered is accounted by these activities, often followed by a resting period before another action of the same intensity occurs [2]. During a top-level soccer match, a player can perform 150 to 250 high-intensity actions [3]. Although high-intensity actions require substantial physiological demands and quickly lead to fatigue [4], trends have shown that in professional soccer, the number of high-intensity actions has risen over the recent decades [5]. This would imply that such actions may adequately discriminate against soccer players at similar physical and psychological levels [6]. Along with the players’ competitive level [7], high-intensity actions completed at maximal levels are associated with the repeated sprint ability (RSA), one of the key determinants in soccer [8,9].

The ability to perform repeated sprints lasting <10 s with short resting intervals between them (<60 s) is crucial for performance in soccer [10]. In practice, RSA can have beneficial effects on the enzymes of the aerobic and anaerobic systems, enhancing the level of phosphofructokinase and myokinase [11] and affecting the increased energy production. Such metabolic changes can be directly associated with explosive strength, sprints and aerobic-related adaptations [8,12]. Despite these advantages, the implementation of RSA in youth players is still lacking [13], possibly because of its specific approach and complexity [14]. Nevertheless, RSA has been constantly proven as a reliable and valid training method for improving a variety of physical performance components in adults and more elite soccer players [15,16].

In general, the RSA incorporates a methodology of linear sprints of <10 s followed by an interval of rest (<60 s) [17]. In regard to linear sprints, change of direction (CoD)-type sprints have become equally important because of their common representation during a soccer match [18]. Specifically, soccer players tend to perform >8 CoD sprint intervals in a minute [19]. Thus, the importance of performing elements with CoDs at a maximal level requires special attention, especially in young soccer players. To date, several studies have examined the effects of an RS–CoD on physical performance in male soccer players [20,21,22,23,24], while only a handful have differentiated between the effects of RSs in a linear direction (RS–LiN) vs. an RS–CoD [13,25,26,27,28]. Results from these studies yielded similar findings, showing no significant differences in the effects of an RS–LiN and RS–CoD in soccer players [25,26,27]. In a study by Sagelv et al. [25], no interaction effect (time × group) in Yo-Yo IR2 or sprint tests was found, which would suggest that both the RS–LiN and RS–CoD produced equal effects on physical performance. Similar observations have been documented in U15, U17 and U19 male soccer players, where both intervention protocols exhibited beneficial changes in the linear and slalom sprint speed, explosive power and maximal aerobic speed [26]. On the other hand, it has been noted that these types of training stimuli performed in a single session per week may not be sensitive enough to produce within-group changes over 8 weeks [27]. For an RS–LiN, unclear variations in sprinting and jumping occurred, while the RS–CoD produced somewhat larger effects, especially in sprinting activities [27]. Therefore, the effect comparison between the RS–LiN and RS–CoD remains inconclusive. Given the fact that implementing RS strategies within the training regime in youth soccer players is challenging, examining separate effects of an RS–LiN and RS–CoD on physical performance would be helpful for strength conditioning coaches to monitor and track fitness levels at individual and group levels.

The main purpose of this study was to examine the effects of a 12-week RS–LiN and RS–CoD training program on physical performance in male U17 soccer players. We hypothesized that the RS–CoD would exhibit larger effects on sprinting-, jumping- and agility-related tests and aerobic capacity in comparison to the RS–LiN.

## 2. Methods

### Study Participants

**Figure 1:**
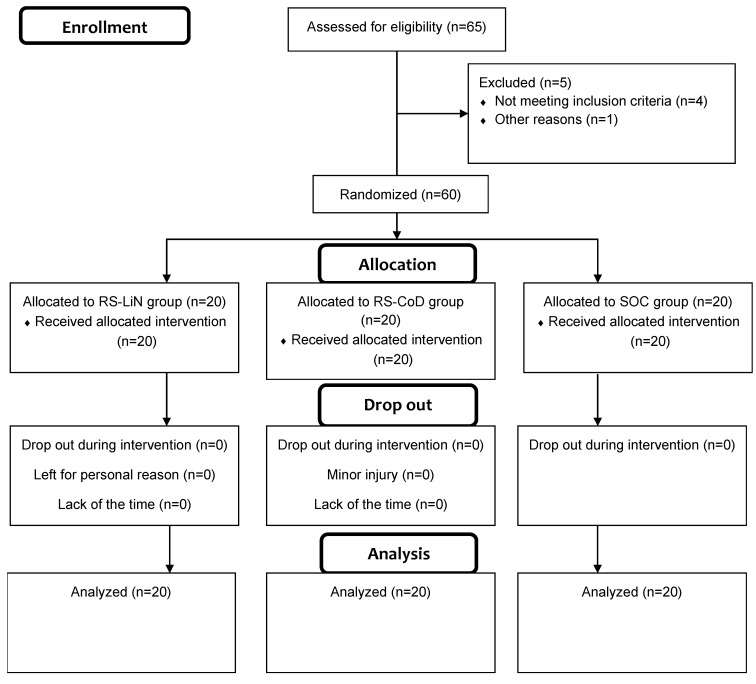
Flow chart diagram of participants’ enrolment, randomization and final analysis.

A flow chart diagram of participants’ enrolment, randomization and final analysis is presented in Figure 1. Male soccer players (age = 16.42 ± 0.21 years; height = 173.32 ± 5.80 cm; weight = 65.19 ± 6.42 kg) from three national-level competitive clubs were recruited in this randomized controlled trial. The G*power sample size calculator indicated that for the a priori analysis of variance for repeated measures (RMANOVA) with a moderate effect size of f = 0.25 [29], a statistical power of 1-β = 0.80, a *p*-value of < 0.05, 3 groups measured at two time points and hypothetical correlations between repeated measures of *r* = 0.50, the minimally required sample size would be 42. To adjust for potential follow-up loss, we increased our final sample to n = 65 soccer players. The inclusion criteria were fully active outfield male soccer players who had experienced no injury or musculoskeletal pain prior to testing and playing soccer for >8 years. The exclusion criteria included a soccer player having an injury-related problem and who failed to attend >85% of training sessions during the competitive season. All data related to inclusion and exclusion criteria were collected by a questionnaire. The final sample included sixty soccer players who met the inclusion criteria and completed the study after being randomly assigned to one of three groups: a change of direction sprint group (RS-CoD; *n* = 20); a linear sprint group (RS-LiN; *n* = 20); and a soccer group performing regular training (SOC; *n* = 20).

Prior to the intervention, all participants trained regularly three days per week in the afternoon hours (between 17:00 h and 19:00 h). A common type of training consisted of a set of technical and tactical components (shooting, dribbling, passing and running with the ball) with the cooperation of the goalkeeper and other players, strength and conditioning parts consisted of small-sided games (5 vs. 5 or 10 vs. 10 soccer players including goalkeepers) and injury prevention exercises were most commonly performed at the end of a training session. For the participants who entered and completed this study, no group differences were present at baseline. The experimental protocol and potential risks were explained to all participants before they provided written consent. All the procedures were anonymous and in accordance with the Declaration of Helsinki. This study was approved by the Ethical Committee Board of the Henan Polytechnic University, Henan Province (Ethical code: HPU-2024-09-4538).

## 3. Intervention Protocol

Based on previous studies, all the study participants completed two familiarization sessions associated with physical performance tests before an intervention in week 0 [24]. During the same week, all participants underwent laboratory and on-field testing procedures. All on-field tests and measurements were completed in a grass soccer field to mimic standardized conditions. The weather conditions were adequate, with an air temperature of 23 °C and a relative humidity of 55%. The laboratory conditions were similar to the on-field conditions, with an indoor temperature of 22 °C and a relative humidity of 53%. We followed the protocol of Sanchez-Sanchez et al. [24], where all participants were required not to eat heavily or drink caffeinated drinks for 2 h and avoid high-intensity aerobic or resistance training 48 h prior to testing. Before initiating the data collection, each participant had a warm-up period of 10 min, which included exercises for dynamic and static stretching, low and high skips and three acceleration and deceleration tasks followed by three 10 m sprints.

We followed the intervention program of Sanchez-Sanchez et al. [24] for the RS–CoD, RS–LiN and SOC groups that lasted for 12 weeks. The SOC group continued the regular soccer training routine, where the RS–CoD and RS–LiN groups completed upgraded soccer training with additional linear or change of direction sprints two times per week (Tuesday and Thursday), totaling 24 sessions. The RS–CoD group performed three sets of ten 15 m sprint intervals lasting <10 s with one CoD of 180° initiated at the end of a track, after which the participants ran at maximal speed to the starting position. Each sprint interval was followed by a recovery period of low-intensity running for <30 s. The resting period between each set was set at 4 min. In total, the weekly training volume was six sets of 10 repetitions over the first 4 weeks, with a progression of 15 repetitions in weeks 5–8 and 20 repetitions in weeks 9–12. The RS–LiN group completed the same number of sprint intervals over a 12-week intervention period, but the participants were instructed to perform linear running on a 31 m track by keeping a constant maximal pace over the entire distance. The reason for the reduced distance in the RS–CoD group is based on recommendations by Stanković et al. [29] where CoD sprints require additional energy and should be compensated for by reducing the distance by 2–3% in comparison to linear sprints.

## 4. Study Variables

*Explosive power* was assessed by two reliable and valid vertical jumps to assess the jump height (cm): (i) the countermovement jump (CMJ) and (ii) squat jump (SJ). We used the Optojump photoelectric cells (Microgate, Bolzano, Italy) to evaluate the flight time (in cm) from take-off to landing phases. The CMJ was performed with the arms on the hips, and based on a pre-recorded signal, each participant flexed their knees to approximately 90° and pushed upward as high as possible without moving the arms from the hips. The same principle was applied to the SJ, but the starting position was from the knees flexed at approximately 90°, and hands were placed on the waist area, and after holding this position for 3 s, the participant was instructed to jump upward vigorously without moving the hands from the hips. Both tests were performed three times with a 4 min rest between each trial. The reliability of all trials for tests was excellent (ICC > 0.85, coefficient of variation [CV] < 2.5%), and the best result was examined in further analyses.

To assess *agility*, we used the T505, 93,639 and 20Y tests. The T505 test involved sprinting to a marked point 5 m away from the starting position, touching the ground with one hand, immediately changing direction with a turn of 180° and running back to the starting point. The 93,639 test was initiated by sprinting over a course of 9 m, after which each player changed direction by 180° and started sprinting for 3 m, making another turn of 180° and running 6 m, continuing with a 180° turn and sprinting for 3 m, before making the last turn of 180° and sprinting over a 9 m course to the finish line. In total, the players covered a distance of 21 m. The final score was recorded as the time (in sec) measured by the photoelectric cell system (Witty, Microgate, Bolzano, Italy). For the 20Y test, two parallel lines were set up in the hall, 10 yards apart, with the center line located exactly halfway between them. Each line was one meter wide. A Witty photocell telemetry system was used for timing (Microgate, Bolzano, Italy), with a pair of cells placed on the center line and a starting position mark 0.5 m from the center line. The participant started from a high start position on an independent signal and repeated the test three times. The movement begins with a sprint to the sideline touched with the foot (covering 5 yards), then a sprint to the other sideline by repeating the same action (covering 10 yards) and finally a sprint back to the center line (covering 5 yards) where the time was stopped. The reliability and factorial validity properties of these tests have been previously confirmed in soccer players [30,31]. Each test included three trials (ICC > 0.80, CV < 3.0%) with a 4 min rest between the attempts, and the best result was considered for further analyses. The aforementioned tests have been illustrated and explained elsewhere [29,30].

To evaluate the *running speed*, the participants completed a set of linear sprinting tests over 5 m, 10m and 20 m [32]. In brief, a photoelectric cell system (Witty, Microgate, Bolzano, Italy) was placed at the starting and finishing lines over a distance of 20 m. Before the test began, the participant took a high standing start position approximately 40 cm behind the starting line. The time began when the participant crossed the first photocell infrared line. During the 20 m sprint section, the participants’ running times at 5 m, 10 m and 20 m were recorded. Similarly to previous tests, three trials were conducted (ICC > 0.90, CV < 2.0%), with the best result being processed in further analyses.

The Running-Based Anaerobic Sprint Test (RAST) was used as a proxy for the anaerobic power output [33]. The test involved completing six 35 m sprints with 10 s rest intervals in between. From a standing start position, each participant sprinted over a course of 35 m. After crossing the 35 m line, the time stopped (and was registered), and the 10 s recovery period began. The same procedure was performed five more times, covering 210 m in total. The test was performed once. The results were presented in sec and maximal power output using the following formula: body mass (kg) * distance covered^2^ * final time^−3^ [33].

The *maximal oxygen uptake* (VO2max) test was conducted using a ramp protocol on a motorized treadmill and determined by a breath-by-breath pulmonary gas exchange system (Quark b^2^, COSMED, Rome, Italy). The starting speed was set at 7 km*h^−1^, and it was increased by 1 km*h^−1^ every 1 min until volitional exhaustion. The criteria for exhaustion included the following: (i) a VO2 of <1.0 mL*kg^−1^*min^−1^ after an increase in speed; (ii) an exchange ratio >1.1; and (iii) a peak lactate of >6 mmol*L^−1^ after exercise [34]. If the participant reported any bodily pain or discomfort, the test was stopped. The recovery period after the exercise included walking at 4.5 km*h^−1^ for 5 min. Aerobic data regarding the VO2max were averaged on a 5 s basis and included as a proxy for the cardiorespiratory capacity in further analyses.

## 5. Statistical Analysis

Basic descriptive statistics are presented as the mean ± standard deviation (SD). Based on previous studies [24], all data were log-transformed to omit the nonuniformity error. A repeated measures analysis of variance (RMANOVA) with pre–post-measurements and the ‘group’ as a between-subject factor was used to examine the main effects for ‘time’ and an interaction term between ‘time’ and ‘group’ (time × group). If significant main effects were observed, a post hoc analysis with Bonferroni corrections was calculated. Within-group differences in pre- and post-measurements were calculated using the Student *t*-test for dependent samples. Cohen’s *D* effect size statistics were used to examine the magnitude of the intervention output with the following threshold values: (i) ≥ 0.2 (small), (ii) ≥ 0.5 (moderate) and (iii) ≥ 0.8 (large) [35]. The significance was set at α < 0.05, and it was two-sided. All analyses were calculated using the Statistical Packages for Social Sciences (SPSS) software program ver. 27 (IBM Corp., Chicago, Il, USA). The raw data of the study participants can be found in the Supplementary Material Table S1.

## 6. Results

The initial data suggested that no differences between the RS-CoD, RS-LiN and SOC groups were observed (*p* = 0.124–0.866). Table 1 shows basic descriptive statistics of the study participants, according to the RS–CoD, RS–LiN and SOC groups. No between-group differences in initial anthropometric characteristics and years of experience were found (*p* > 0.05).

Within-group changes in physical performance are presented in Table 2. The significant and most beneficial improvements were found in the RS–CoD group. Soccer players assigned to this group displayed large increases for the T505 (mean diff. = 0.13 s, 95% CI 0.10–0.16); SJ (mean diff. = 2.03 cm, 95% CI 1.37–2.70); 20Y (mean diff. = 0.21 s, 95% CI 0.17–0.25); sprint for 5 m (mean diff. = 0.09 s, 95% CI 0.05–0.12), 10 m (mean diff. = 0.13 s, 95% CI 0.08–0.17) and 20 m (mean diff. = 0.15 s, 95% CI 0.11–0.19); and RAST (mean diff. = 0.25 s, 95% CI 0.19–0.31) and moderate increases for the 93639 (mean diff. = 0.13 s, 95% CI 0.12–0.15), CMJ (mean diff. = 1.77 cm, 95% CI 1.15–2.39), RAST power output (mean diff. = 33.17 W, 95% CI 22.21–44.12) and VO2max (mean diff. = 1.78 mL * kg^−1^ * min^−1^, 95% CI 0.57–3.00). For the RS–LiN group, the largest improvement was observed for the 10 m sprint (mean diff. = 0.10 s, 95% CI 0.06–0.13), while 93639 displayed the least beneficial improvement (mean diff. = 0.09 s, 95% CI 0.08–0.10). In the SOC group, small to moderate ESs for physical performance were shown, where T505 (mean diff. = 0.05 s, 95% CI 0.04–0.07) and 20Y (mean diff. = 0.12 s, 95% CI 0.10–0.14) exhibited the largest increases, opposed to other variables.

The between-group differences are presented in Table 2. The RS–CoD group exhibited significantly beneficial effects in the 93639 (*F*_2,59_ = 23.79, *p* < 0.001), SJ (*F*_2,59_ = 18.12, *p* < 0.001), CMJ (*F*_2,59_ = 6.73, *p* = 0.002), 20Y (*F*_2,59_ = 10.66, *p* < 0.001), 5 m sprint (*F*_2,59_ = 8.92, *p* < 0.001) and 20 m sprint tests (*F*_2,59_ = 22.23, *p* < 0.001), compared to the RS–LiN and SOC groups. Opposed to the SOC group, the RS–CoD group showed significantly larger changes in the T505 (*F*_2,59_ = 11.10, *p* < 0.001), 10 m sprint (*F*_2,59_ = 9.21, *p* < 0.001), RAST (*F*_2,59_ = 12.58, *p* < 0.001), RAST power output (*F*_2,59_ = 5.86, *p* = 0.005) and VO2max tests (*F*_2,59_ = 3.56, *p* = 0.035). The RS–LiN group performed better in the 93639 test, sprints at 10 and 20 m and RAST (*p* < 0.05) than the SOC group.

## 7. Discussion

The main purpose of this study was to examine whether RS–LiN and RS–CoD intervention programs would have beneficial effects on physical performance in male U17 soccer players. Findings suggest that both groups enhanced their physical performance over 12 weeks, although the RS–CoD group seemed to outpower the RS–LiN and SOC groups with more beneficial effects, especially in sprinting activities.

To date, only a handful of studies have simultaneously compared the effects in linear vs. change of direction forms in male soccer players [13,25,26,27,28]. In a 30-week intervention study, both RS–CoD and RS–LiN programs exhibited positive effects on cardiorespiratory and neuromuscular physical performance in highly trained soccer players [26]. Although the within-group differences were small to moderate, the between-group differences in pre–post-changes yielded non-significant main effects, indicating that both training interventions produced similar improvements over time [26]. Similar observations were presented in a 22-week intervention study, which compared the effects of the RS–CoD and RS–LiN for Yo–Yo IR2, sprints over 10 m and 20 m and VO2max [25]. The mentioned study showed that all male soccer players, irrespective of group, increased their Yo–Yo IR2 by 9.1%, but the ‘time × group’ interaction was not significant (_p_η^2^ = 0.06). Interestingly, no improvements in the sprints over 10 m and 20 m and VO2max were found [25]. In a study by Beato et al. [27], a single session of straight line vs. change of direction sprint intervals failed to lead to physical performance improvements in explosive power; RSA; or sprints over 10 m, 20 m and 40 m. Also, the main effects between RS–CoD and RS–LiN groups showed that both intervention programs exhibited no between-group differences [27]. Sanchez–Sanchez et al. [24] reported that the SOC group achieved greater improvements in aerobic capacity and sprint decrements during RS ability tests, compared with the RS-COD groups. As expected, the RSA–CoD group improved their RSA and change of direction performance, opposed to the SOC group [24]. Another study including a 6-week intervention protocol consisted of linear and change of direction sprints in female soccer players showed no significant ‘time x group’ main effects for aerobic, musculoskeletal and agility performance, although the group that performed change of direction sprint intervals exhibited larger improvements in sprints over 30 m, compared to the linear group [29]. In general, differences in pre–post-changes for physical performance between RS–CoD and RS–LiN groups remain non-significant, with similar improvements between the two intervention protocols, which is not in line with our study. Since we showed that the RS–CoD group exhibited greater effects in all physical performance tests in comparison to the RS–LiN and SOC groups, we could assume that the increased training stimuli with different movement patterns and volumes over 12 weeks led to larger improvements. Indeed, the training load in terms of the number of sprints was inconsistent between the studies. For example, the post-intervention number of sprints in this study was 720 over 12 weeks, while a study by Pavillon et al. [26] reported a total of 1200 sprints over 30 weeks, which would produce a different sprint/week ratio. Additionally, soccer players in the RS–CoD group were unfamiliar with CoD sprint intervals, which might have led to greater effects. This is not surprising, since evidence suggests that the CoD sprint intervals decrease with age, where younger soccer players (U15) may excel at shorter sprints and CoDs at lower speeds, while older players (U18) can demonstrate better linear sprinting abilities and power at longer distances [26]. Thus, the CoD sprint training should be initiated from an early age, but the effects are expected to be normalized by genetic predispositions [36].

The difference between CoD and linear sprints should be observed with neuromuscular and metabolic demands [19,37]. Linear sprinting primarily focuses on maximizing horizontal velocity, by changing kinematical characteristics of running through the step/stride length and pace. On the other hand, CoD sprint intervals are accompanied by the acceleration–deceleration dynamics associated with a greater engagement of muscle antagonists (eccentric contraction properties) [38]. Such actions may lead to oxidative stress faster and increases in muscle damage following soccer training [39]. Opposed to linear sprinting, running with CoDs also increases energy demands, requiring greater blood lactate concentrations, heart rates and aerobic capacity [13]. As we highlighted, the more beneficial effects of CoD sprint intervals may be attributed to the high training volume across 12 weeks, as soccer players performed 720 sprint intervals, opposed to lower training volumes in other studies [26]. Unfortunately, the true energy demand of the CoD with a turn of 180° has been rarely investigated, as the speed or distance are not adjusted for the time lost during the turns, limiting the possibility of actual CoD effects on physical performance in soccer players [19].

Irrespective of a relatively high heterogeneity between the studies regarding comparisons between CoD and linear sprint intervals on physical performance, the findings of this study confirmed that sprints with a CoD of 180° may be more beneficial for physical performance improvements, as CoD movements mimic more specific movements similar to soccer. The practical implications of RSs with CoDs performed during the pre-season represent good training stimuli for physiological and performance-related outcomes. Therefore, such a training approach should be implemented within regular soccer training for additional effects, especially for sprinting activities. Because the training itself took approximately 20 min to complete, strength and conditioning coaches can consider CoD sprint intervals with a turn of 180° as a pragmatic strategy for positive increases in soccer-specific performance tests. Since these tests have been previously described as reliable and valid, the intervention protocol used in this study may be a better alternative to linear sprinting activities, due to greater changes in physical performance.

This study is not without limitations. Even though we observed significant positive effects in the RS–CoD, RS–LiN and SOC groups for physical performance, the intervention duration of 12 weeks was arbitrary chosen, and due to time restrictions, we were unable to prolong the intervention process to >20 weeks, as described in previous studies [25,26]. By performing the intervention over more weeks, it would be possible to examine the adaptation process to the RS–CoD intervention and to assume the best intervention length that would produce the greatest positive changes on physical performance. Since the research around CoD sprint intervals, training loads, durations, energy demands and effectiveness is still relatively limited, although promising, our findings should be interpreted with caution. Second, the CoD sprint interval performance has been shown to decline with age [19,26], and we were unable to examine age-related differences following RS–CoD and RS–LiN interventions. Third, no invasive methods were performed to collect blood samples, which we speculated to be correlated with changes produced by CoD or LiN sprint intervals. Nevertheless, this is one of the first studies that compares the effects of the RS–CoD and RS–LiN opposed to SOC training in U17 soccer players [40].

## 8. Conclusions

Based on the obtained findings, we may conclude that different types of training (RS–CoD, RS–LiN and SOC) produce beneficial and positive effects on physical performance in young soccer players. However, the largest effects are observed for the CoD sprint intervals, where the RS–CoD group exhibited more beneficial improvements in all physical performance tests, especially for sprinting activities, compared to the RS–LiN and SOC groups. Thus, this study can be considered as a good starting point to enhance or even maintain high physical performances in the pre-season period in U17 male soccer players.

## Figures and Tables

**Table 1:** General information of the study participants, according to groups.

Variables	Total (*n* = 60)	RS–CoD (*n* = 20)	RS–LiN (*n* = 20)	SOC (*n* = 20)	*p*-Value
	Mean ± *SD*	Mean ± *SD*	Mean ± *SD*	Mean ± *SD*	
Age (years)	16.42 ± 0.21	16.38 ± 0.24	16.46 ± 0.20	16.41 ± 0.19	0.856
Height (cm)	173.32 ± 5.80	172.03 ± 5.48	173.95 ± 6.13	173.98 ± 5.86	0.482
Weight (kg)	65.19 ± 6.42	64.83 ± 5.01	64.44 ± 7.85	66.29 ± 6.27	0.638
BMI (kg*m^−2^)	22.29 ± 2.90	22.51 ± 1.32	21.88 ± 2.69	22.48 ± 1.41	0.501
Experience (years)	10.21 ± 1.25	10.30 ± 1.23	10.11 ± 1.31	10.22 ± 1.26	0.728

BMI: Body mass index; *p* < 0.05.

**Table 2:** Physical performance in RS-CoD, RS-LIN and SOC groups before and after 12-week training intervention.

Variables	Pre	Post	∆ (%)	ES	*p*-Value
**RS–CoD (*n* = 20)**	Mean ± *SD*	Mean ± *SD*			
T505 (s)	2.54 ± 0.12	2.41 ± 0.10	−5.12% ^b^	1.18	**<0.001**
93639 (s)	7.74 ± 0.33	7.60 ± 0.33	−1.81% ^a,b^	0.42	**<0.001**
SJ (cm)	26.17 ± 2.18	28.20 ± 2.49	7.76% ^a,b^	0.87	**<0.001**
CMJ (cm)	28.13 ± 3.01	29.90 ± 3.34	6.29% ^a,b^	0.56	**<0.001**
20Y (s)	4.62 ± 0.20	4.41 ± 0.20	−4.55% ^a,b^	1.05	**<0.001**
Sprint, 5 m (s)	1.23 ± 0.09	1.14 ± 0.06	−7.32% ^a,b^	1.18	**<0.001**
Sprint, 10 m (s)	2.04 ± 0.11	1.92 ± 0.08	−5.88% ^b^	1.25	**<0.001**
Sprint, 20 m (s)	3.48 ± 0.11	3.33 ± 0.10	−4.31% ^a,b^	1.43	**<0.001**
RAST (s)	6.16 ± 0.20	5.91 ± 0.18	−4.07% ^b^	1.31	**<0.001**
RAST power output (W)	306.14 ± 32.30	339.31 ± 30.78	10.83% ^b^	0.73	**<0.001**
VO2max (mL * kg^−1^ * min^−1^)	50.45 ± 3.15	52.23 ± 3.78	3.53% ^b^	0.51	**0.006**
**RS–LiN (*n* = 20)**					
T505 (s)	2.47 ± 0.15	2.38 ± 0.16	−3.64%	0.58	**<0.001**
93639 (s)	7.71 ± 0.50	7.62 ± 0.49	−1.12% ^c^	0.18	**<0.001**
SJ (cm)	26.44 ± 2.05	27.26 ± 2.40	3.10%	0.37	**<0.001**
CMJ (cm)	28.06 ± 2.39	29.01 ± 2.45	3.39%	0.39	**<0.001**
20Y (s)	4.62 ± 0.23	4.47 ± 0.22	−3.25%	0.67	**<0.001**
Sprint, 5 m (s)	1.23 ± 0.06	1.20 ± 0.06	−2.44%	0.50	**0.016**
Sprint, 10 m (s)	2.06 ± 0.09	1.96 ± 0.12	−4.86% ^c^	0.94	**<0.001**
Sprint, 20 m (s)	3.50 ± 0.14	3.40 ± 0.14	−2.86% ^c^	0.71	**<0.001**
RAST (s)	6.18 ± 0.24	6.01 ± 0.28	−2.75% ^c^	0.65	**<0.001**
RAST power output (W)	343.57 ± 46.91	370.04 ± 48.51	7.70%	0.55	**<0.001**
VO2max (mL * kg^−1^ * min^−1^)	50.03 ± 2.57	51.01 ± 2.80	1.96%	0.36	**0.003**
**SOC (*n* = 20)**					
T505 (s)	2.49 ± 0.13	2.43 ± 0.10	−2.41%	0.52	**<0.001**
93639 (s)	7.87 ± 0.52	7.81 ± 0.52	−0.76%	0.12	**<0.001**
SJ (cm)	26.12 ± 1.91	26.46 ± 1.93	1.30%	0.18	**<0.001**
CMJ (cm)	27.45 ± 1.60	28.15 ± 1.62	2.55%	0.43	**<0.001**
20Y (s)	4.67 ± 0.22	4.55 ± 0.21	−2.57%	0.56	**<0.001**
Sprint, 5 m (s)	1.22 ± 0.10	1.20 ± 0.09	−1.64%	0.21	0.160
Sprint, 10 m (s)	2.01 ± 0.09	1.99 ± 0.07	−1.00%	0.25	0.081
Sprint, 20 m (s)	3.49 ± 0.14	3.45 ± 0.14	−1.15%	0.29	**<0.001**
RAST (s)	6.13 ± 0.22	6.05 ± 0.23	−1.31%	0.36	**<0.001**
RAST power output (W)	309.46 ± 36.26	324.93 ± 38.07	5.00%	0.42	**<0.001**
VO2max (mL * kg^−1^ * min^−1^)	50.44 ± 2.88	50.72 ± 2.89	0.56%	0.10	**<0.001**

T505: 5-0-5 agility test; 93639: agility test 93639 m; SJ: squat jump; CMJ: countermovement jump; 20Y: 20-yard agility test; RAST: running-based anaerobic sprint test; VO2max: maximal oxygen uptake; ^a^ denotes significant difference between the RS-LiN and RS-CoD groups (*p* < 0.05); ^b^ denotes significant difference between the RS-CoD and SOC groups (*p* < 0.05); ^c^ denotes significant difference between the RS-LiN and SOC groups (*p* < 0.05); and *p* < 0.05.

## Data Availability

The datasets used and/or analyzed during the current study are available from the corresponding author on reasonable request.

## Supplementary Materials

The following supporting information can be downloaded at https://www.mdpi.com/article/10.3390/sports14020044/s1, Table S1: Raw data of study participants.
